# The dataset on fear of failure, entrepreneurship education, psychological and contextual predictors of entrepreneurial intention

**DOI:** 10.3389/fpsyg.2022.954285

**Published:** 2022-08-17

**Authors:** Imran Anwar, Prabha Thoudam, Mohammed Samroodh, Maebeama Thoudam, Imran Saleem

**Affiliations:** ^1^University Centre for Research and Development, Chandigarh University, Mohali, India; ^2^Delhi School of Professional Studies and Research, New Delhi, India; ^3^Department of Commerce, Aligarh Muslim University, Aligarh, India; ^4^Department of Commerce, Dhanamanjuri University, Imphal, India; ^5^University of Buraimi, Buraimi, Oman

**Keywords:** entrepreneurial intention, entrepreneurship education, entrepreneurial motivation, perceived cultural support, fear of failure

## Introduction

As a consequence of the shifts brought about by growing globalization, a more diverse labor force, and fast technological advancements, workplaces and organizational structures have transformed. This transformation has changed individuals' mindsets about entrepreneurship as a career choice (Sullivan and Baruch, 2009; Anwar et al., 2022) and given entrepreneurship a nod over other employment alternatives (Edelman et al., 2016). As a result, entrepreneurship became one of the most talked-about job possibilities for the younger generation. Governments and educational institutions are putting more emphasis on fostering entrepreneurship. Student entrepreneurship is also being studied as an alternative to the creation of entrepreneurial ambitions by researchers (Anwar et al., 2020; Hassan et al., 2020). Many people have entrepreneurial goals, but research shows that only a small percentage follow through with their plans. Understanding the elements that contribute to entrepreneurial intentions and how these factors might translate intentions into behavior is becoming more important.

Due to their proactive and inventive response to economic and social difficulties, entrepreneurs are recognized as the driving force behind change. When the business climate is bad, it might affect them as well. India is encouraging its youth to become self-reliant to promote entrepreneurship in the country by pursuing a career in entrepreneurship and has lately introduced a program called “AATMANIRBHAR BHARAT” with the slogan “vocal for local,” which is a reference to the country's current employment crisis. An estimated 33.3% of individuals in India have expressed a desire to start their own business, but just 15% have followed through on this desire by actually starting their own business (GEM Global Report, 2020). The relationship between intention and behavior has been proven to be substantially connected. In their research, Ajzen et al. (2009) concluded that intention and actual behavior are closely connected, with intention being the strongest predictor of actual behavior. As a result, the ability to translate intentions into real action is determined by how strong and consistent the intentions are, which are influenced by the motivations that underpin entrepreneurship activities. Psychological and environmental variables play an important part in establishing these motives, which push a person toward starting a business (Hassan et al., 2021). The “entrepreneurial event model” by Shapero and Sokol (1982) and the “theory of planned behavior” by Ajzen (1991) are the two primary conceptual frameworks that have been dominantly utilized to describe the predecessors and relevance of entrepreneurial intents. However, empirical research has revealed a gap between these models and the practicality of entrepreneurship in today's environment (Barba-Sánchez and Atienza-Sahuquillo, 2018), as there are a number of other cognitive and psychological (viz. entrepreneurial motivations, fear of failure, entrepreneurship education) and contextual factors (viz. perceived cultural support, perceived government support, and access to entrepreneurial) that all play a role. Accordingly, this study builds on these psychological and contextual determinants of entrepreneurial intention and gathers primary data using questionnaires.

## Methods

### Participants

This study furnishes survey-based primary data on seven cognitive, psychological and contextual latent variables meant to capture the phenomenon of nascent entrepreneurial intention among the students of three different Indian universities. The data were collected in February 2021 using Google Forms. Based on the recent literature in the domains of entrepreneurial intention, student entrepreneurship, and entrepreneurship education, UG and PG level students studying business and management courses in Indian universities were chosen as the target sample of the study. Three Indian universities, namely, University of Kashmir, Aligarh Muslim University, and Jamia Hamdard University Kannur Campus, were selected for data collection.

### Survey instrument

A questionnaire (consisting of 33 manifest variables) was developed for data collection, comprising two sections. The first section was designed to measure seven latent constructs (viz., perceived cultural support, government support policies, entrepreneurial intention, access to entrepreneurial finance, entrepreneurship education, and fear of failure) using multi-item measurement scales. The second section of the questionnaire was designated to capture respondents' demographic attributes, viz. age (three categories), gender (two categories), level of education (two categories), and university name (three categories). The questionnaire was designed borrowing published and validated measurement scales duly citing their sources. Two five-item scales to measure entrepreneurial intention and entrepreneurship education were taken from Liñán and Chen (2009). We cited Solesvik (2013) for adopting the scale for measuring entrepreneurial motivation (five items). The scale to capture government support policies (three items), the study of Gnyawali and Fogel (1994), was duly cited. Further, we took two five-item scales for gauging perceived cultural support and fear of failure from Liñán et al. (2020) and Cacciotti et al. (2020). Lastly, we measured access to entrepreneurial finance using a five-item scale developed by Matshekga and Urban (2013).

### Pilot survey

Prior to moving ahead with the pilot and main survey, the questionnaire items were first put to the screening for subjectivity and language accuracy checks. The questionnaire was sent for qualitative and language accuracy check to four university professors/academicians with expertise in the teaching and research of entrepreneurship. Their suggestions regarding subjectivity and unidimensionality of the scales were incorporated to enhance the soundness of the questionnaire. Following the qualitative remedial suggestions of Podsakoff and Organ (1986), the language of each questionnaire item was also made clear, unambiguous, single-faceted, and error-free, avoiding double-barreled questions. The introductory part of the questionnaire narrated the purpose of the survey along with ensuring the respondents about the confidentiality and anonymity of their responses. However, the survey did not ask for any such information such as; name, address, email, etc., that might lead to personal identification. Further, a pilot survey was run to ensure the internal consistency and reliability of the measurement scales (using Alpha reliability), taking a sample of 60 students (20 from each sampled university).

### Final survey

Following the pilot survey, the main survey was conducted, and the data were collected from three different universities in India, namely; University of Kashmir, Aligarh Muslim University, and Jamia Hamdard University Kannur Campus. Using Google Forms, the UG and PG students from Business and Management backgrounds were contacted to fill out the questionnaire in February 2021. A total of 450 questionnaires (150 in each university) were administered, and 354 completed questionnaires were received (at a retrieval rate of 78.67%), which were then processed for cleaning and screening before establishing the measurement model (reliability and validity analysis) using confirmatory factor analysis in AMOS.

### Data screening

The sample of 354 responses suffered from some unengaged and outlier responses; hence it was screened and cleaned before proceeding with further statistical validation analyses. Firstly, the data were examined for unengaged and improper responses, and it was found that 13 respondents did not get engaged while responding to the questionnaire; hence these responses were deleted from the sample. Secondly, using Cook's distance method, statistical outliers were detected in the sample data. A response with Cook's statistics >1 is said to be an outlier (Stevens, 2012). During the process, 12 responses produced Cook's statistics >1; hence they were termed potential statistical outliers and removed from the dataset, leaving a final sample of 329 responses (see Table 1 for the demographic profile of the respondents).

**Table 1:** Demographic profile of the sample (*N* = 329).

**Variable name**	**Category**	**Frequency (*N*)**	**Percentage (%)**
Age	<22 Years	174	52.9
	22–25 Years	121	36.8
	Above 25 years	34	10.3
Gender	Male	171	52.0
	Female	158	48.0
Level of education	UG	195	59.3
	PG	134	40.7
University	University of Kashmir, Srinagar	109	33.1
	Aligarh Muslim University, Aligarh	117	35.6
	Jamia Hamdard University Kannur Campus	103	31.3

Lastly, the data were also checked for statistical method bias using Harman's single factor method suggested by Podsakoff and Organ (1986). All 33 manifest variables were forced to load on a single component (using Principal Component Analysis for extraction and Varimax for rotation) to account for the total explained variance. The results showed that all 33 manifest variables could explain a total variance of 33.195%, which is far below the cut-off limit of 50%, affirming that the data are not suffering from method bias.

## Results

Global fitness, validity (convergence and divergence), and reliability (scale's internal consistency) of the measurement model were ensured by running a covariance-based CFA model in AMOS v.21. A reflective CFA model, with 33 observed items converging with seven latent constructs, was drawn and run to achieve model's global fit indices and standardized factor loadings of observed items.

### Model fit and convergent validity

The results from the CFA model confirmed that the model holds a very good fit, evidencing model fit indices fall in the good and excellent categories (see Table 2). Convergent validity of the data was established, taking into consideration standardized CFA loadings of observed items and the average variance extracted (AVE) value for each latent construct. Convergent validity is said to be met when the AVE value is >0.50, which refers to the squared value of the average CFA loading of a latent construct (Bagozzi and Yi, 1988; Hair et al., 2006). The results, shown in Table 3, evidence that AVE values for each latent construct are well above the threshold, thereby ensuring the convergent validity of the data. Moreover, the data were also tested for scale reliability for each latent construct using Cronbach's Alpha (α) and Composite Reliability (CR) statistics. A construct is considered to meet scale reliability criteria if α and CR statistics are >0.70 (Hair et al., 2006). Statistics for α and CR are found well above the threshold of 0.70, thus meeting the benchmark of scale reliability.

**Table 2:** CFA model fit indices, convergent validity, composite and Cronbach's Alpha reliability.

**Model**	**CMIN/DF**	**GFI**	**CFI**	**TLI**	**RMSEA**
Study model	1.932	0.905	0.938	0.930	0.053
Recommended value	Acceptables 1–4	>0.90	>0.90	>0.90	<0.07
	Wheaton et al. (1977)	Shevlin and Miles (1998)	Shevlin and Miles (1998)	Hu and Bentler (1999)	MacCallum et al. (1996)
**Construct name**		**Avg CFA loading**	**AVE**	**CR**	**Cronbach's Alpha (α)**
Perceived cultural support		0.775	0.601	0.882	0.881
Government support policies		0.743	0.552	0.786	0.782
Entrepreneurial intention		0.841	0.707	0.923	0.921
Access to entrepreneurial finance		0.758	0.575	0.871	0.869
Entrepreneurship education		0.735	0.540	0.840	0.842
Entrepreneurial motivation		0.838	0.702	0.921	0.921
Fear of failure		0.739	0.546	0.854	0.866

**Table 3:** Correlations, divergent validity, and descriptive statistics.

**Construct name**	**PCS**	**GSP**	**EI**	**Fin**	**EE**	**EM**	**FoF**
PCS	**0.775**						
GSP	0.656	**0.743**					
EI	0.363	0.312	**0.841**				
Fin	0.676	0.693	0.306	**0.758**			
EE	0.542	0.452	0.430	0.487	**0.717**		
EM	0.495	0.425	0.661	0.381	0.534	**0.838**	
FoF	0.240	0.172	−0.119	0.328	0.130	−0.085	**0.739**
Mean	4.903	4.575	5.340	4.643	5.221	5.462	4.153
SD	1.276	1.413	1.662	1.318	1.187	1.494	1.569
Skewness	−0.346	−0.368	−0.974	−0.280	−0.368	−1.092	−0.190

*Squared root of AVE has been shown in bold on diagonals, and it should be greater than off-diagonal values for divergent validity. PCS, Perceived Cultural Support; GSP, Govt. Support Policies; EI, Entrepreneurial Intention; Fin, Access to Entrepreneurial Finance; EE, Entrepreneurship Education; EM, Entrepreneurial Motivation; FoF, Fear of Failure*.

Following Fornell and Larcker's approach, the authors ensured the discriminant validity of the data by comparing the squared root value of AVE (on-diagonal bold values in Table 3) of each construct to off-diagonal correlation coefficients. Discriminant validity among the latent variables persists when on-diagonal bold value for each construct is greater than its correlations (off-diagonal values) with other latent variables (Fornell and Larcker, 1981). Table 3 also reports descriptive statistics (mean and SD) for each latent variable along with skewness statistics for confirming the multivariate normality assumption (Kline, 1998). Skewness statistics for each latent variable are found within the range of −2 and +2 hence inferring that the data hold multivariate normality (Kline, 1998).

## Value and use of the data

This study furnishes the unique primary survey-based data to understand the causal effect of cognitive and psychological factors (viz. entrepreneurship education, entrepreneurial motivation, fear of failure) and contextual factors (viz. perceived cultural support, Govt. support policies, access to entrepreneurial finance), entrepreneurial intention of university students from India. This study also provides multidimensional data on fear of failure, which is very scant in the literature on fear of failure as the majority of the studies have only used a single-item scale to measure fear of failure. These data can be useful for the researchers studying nascent and student entrepreneurship as it contains the data on cognitive, psychological, and contextual predictors of entrepreneurial intention. Moreover, the findings from this data can also be of practical use for the universities and policymakers in designing entrepreneurship course curricula and promoting entrepreneurship among university students. As the data are metric/quantitative in nature and have been collected using reflective scales, therefore, they can be used for advanced-level modeling like mediation, moderation, and moderated mediation. Almost equal sample distribution between male and female students also offers a possible model comparison between male and female students using multi-group SEM.

## Data availability statement

The datasets presented in this study can be found in online repositories. The names of the repository/repositories and accession number(s) can be found below: Mendeley data https://data.mendeley.com/datasets/mv22t9c55p/2.

## Ethics statement

The studies involving human participants were reviewed and approved by Institutional Ethics Committee, Aligarh Muslim University. The patients/participants provided their written informed consent to participate in this study.

## Author contributions

IA conceptualized the study, wrote the original draft, and performed the statistical analysis. PT took care of data curation, methodology, and acquired funding. MS and MT conducted the survey and reviewed and edited the final draft. IS supervised, administered the project, and contributed to editing the final draft. All authors contributed to the article and approved the submitted version.

## Conflict of interest

The authors declare that the research was conducted in the absence of any commercial or financial relationships that could be construed as a potential conflict of interest.

## Publisher's note

All claims expressed in this article are solely those of the authors and do not necessarily represent those of their affiliated organizations, or those of the publisher, the editors and the reviewers. Any product that may be evaluated in this article, or claim that may be made by its manufacturer, is not guaranteed or endorsed by the publisher.
